# 

**Published:** 1870-01

**Authors:** 


### INDEX TO VOL. XI.

PAGE.
Abdominal Cavity, Inflammation within, by F. K. Bailey, M.D., 129
Address by J. H. Brewer, M.D. 104
Address of Prof. J. S. Sherman, 241
Address before American Association of Medical Editors, by N. S. Davis, M.D., 413
Aggregation in the Dublin Lying-in Hospital, 253
A New Year and a New Volume 51
Anæsthesia, Relative Danger of, by Chloroform and Ether. By Prof. E. Andrews, A.M., M.D., 259
Anatomy. By Henry Gray, F. R.S., and T. Holmes, M.A., 387
Aphonia, New Treatment for, 636
Archives de Physiologie, A. Book, 51
Arts, The, Published by Jos. M. Hirsh & Co., Chicago, 183
Association, Alumni, 179, 239
Association, American Medical, 59, 184, 390, 712
Aspirateur Souscutane, Dr. Dieulafoy's, 245, 392
Atropia *versus* Opium. By Theodore Griffin, M.D., 488
Antiseptic, A New, 759
Amputations in the Pennsylvania Hospital, 767
Abdomino-Vaginal Impalement, 768
Anatomical Specimens, Preservation of, 771

Bromides, the. By F. K. Bailey, M.D., 19
Bright's Disease, Pathology of. By W. B. Lewis, M.D., 50
Biliary Calculi, Convulsions and Death. J. A. Ballard, M.D., 135
Bright's Disease, Milk Diet and Treatment of, 700
"Bitters," The Sale of, 316
Board of Public Works, Report, 451
"Bridge Cases," Pathology of the, 517
Bromides, The. By Z. McElroy, 584
Bromide of Potassium, its Effects on Children, 638
Biliary Calculi, Chloroform in Treatment of, 649
Bloodvessels, Minute History of, 702
Bromide of Lithium, The Use of, 764

PAGE.
Chloroform, Death from, 59
Chloroform, almost a Death from, 397
Copper, Effects of on the System, 60
Coccyodynia, Case of. By W. R. Fox, M.D., 76
Consumption, Climate for Cure of, 118
Congenital Syphilis. By W. H. VanBuren, M.D., 115
College Commencement, 179
Catarrh and Bronchitis, Treatment of. By G. Johnson, M.D., 167
Chicago Medical College Commencement, 246
Clinical Medicine and Physical Diagnosis, Manual of. By T. H. Tanner, M.D., 245
Cell Doctrine. By J. Tyson, M.D., 244
Children, Diseases of, 245
Conjugal Onanism. By L. F. E. Bergeret, 310
Chicago Medical College, 313, 366, 455, 586
Cynauche Tonsillaris, 397
Cinchona, 462
Chloral, 460, 513 628
Cerebro Spinal Meningitis. By D. A. Sheffield, M.D., 485
Copper as a Preventive for Cholera, 522
Chloroform, Use of in Ophthalmic Surgery. By H. W. Boyd, M.D., 554
Carbolic Acid, Double Salts of, 634
Compendium of Medical Science, 640
Correction—New Work 641, 776
Cervical Pneumogastrics, 697
Cardiac Disease, Rare Form of. By N. S. Davis, M.D., 677
Catarrh, A New Remedy for, 683
Cancer of Uterus, 696
Clinical Reports—Clinic of Prof. Davis, 145, 284, 705
Croup, Treatment of, 704
Carbolic Acid, Poisoning by. By T. G. Roddick, M.D., 715
Cervical Vertebræ, Dislocation of Three, Reduction and Recovery, 769

Diploma Venders, 52, 146, 182
Diplomas, Bogus, 124

WI
CH56

PAGE.
Dislocations, Compound. By Jos Lister, F.R.S., 578
Diabetes Mellitus, 700
Essays, Four Prizes for, 55
Epilepsy, Treatment for, 59, 462
Exuberance of Life in Great Varieties in Ocean's Depths, 186
Epileptiform Convulsions. By Brown-Sequard, 137
Enuresis, Collodion in, 396
Epilepsy. By Brown-Sequard, 404
Ergot, 459, 461
Endometritis, Uterine Injections in, 521
Explanation, 585, 711
Exchanges, 711
Eczema, 715
Erigeron as a Hemostatic, Tincture of, 751

Foreign Items, 53
Fragment of Knife-blade Lodged in Chest. By J. F. Snyder, M. D., 401
Fatty Degeneration, 700

Gums, Scarification in Dentition, 41
Gynæcological Society of Boston, 583
Gelseminum, Therapeutic Effects of, 770

Hip Dislocations. Reply of Prof. Bigelow to Prof. Gunn, 25
Hospital Notes, Reports, etc., 33, 688
Homœopathy, Modern, 55
Hydrophobia, Indian Hemp in, 186
Humanity, Oldest Relic of, 188
Heart, Diseases of. By Austin Flint, M.D., 311
Humbolt Medical College, 366
History of Medicine, A Chair of, 462
Hemp, American, Value of, 650
Humulus Lupulus in Intermittent Fever, 639
Heart, Prognosis in Chronic Diseases of the, 629
Heath's Practical Anatomy, 709
History of Minute Bloodvessels, 702

Intraocular Tumors, a Treatise on. By H. Knapp, M.D., 50
Infants and Children, Diseases of. By Eustace Smith, M.D., 178
Intemperance, 253
Illinois State Medical Society, 246, 249, 312, 490
Iodine in Goiture, 238
Indigestions. By T. K. Chambers, 311

PAGE.
Insanity. By F. K. Bailey, 385
Indiana State Medical Society, 425
Insane, Asylum Schools for the, 769
Intra-Uterine Medication, 761

Jefferson Medical College, 368
Joints, 635, 699

Kansas Medical Law, 249
Kidney, Excision of, 396

Letter from Vienna. By F, 95
Letters from F. K. Bailey, M.D., 149, 443
Locomotor Ataxy, A Lecture. By Meredith Clymer, M.D., 152
Letter by F., 213
Liver, Glycogenic Function of, 458
Licences, Report in Relation to, 506
Liver, Cancer of. By Prof. Davis, 529
Lachrymal Obstructions. By H. W. Boyd, M.D., 593
Limbs, Reproductions of, 646
Letter from Theo. Griffin, M.D., 624
Letter from E. Holderness, 684
Liver, Encephaloid Cancer of the. By T. G. Williams, 686
Lecture, Introductory. By H. A. Johnson, M. D., 657
Locomotor Ataxy, Progressive. Clinic by Professor Davis, 752
Medical College Education, 52
Medical College Convention, 54, 180, 353, 377, 383
Mortality Report, 56, 125, 189, 251, 315, 391, 457, 525, 587, 651, 717, 777
Medical "Wrinkles," 462
Mechanical Support during Labor, 445
Medical Society, Chicago, 180, 312, 438, 523, 565, 746, 749
Medical Society, Allen County, 390
Medical Society, Transactions of, State of New York, 387
Medical Societies, Proceedings of, 388
Mercy Hospital, 140. 439
Mental and the Physical. By F. C. March, M. D., 65
Medical Colleges in Chicago, 356, 641, 645
Medical College of S. Carolina, 372
Medical Department of Harvard University, 361
Medical Department of Williamette University, 367
Medical Institution of Yale College, 361

PAGE.
Miami Medical College, ---------- 371
Morphia, ---------------------397, 461
Medical Ethics, ----------------- 121
Military Tract Medical Association, Report of, ------------ 102
Minnesota State Medical Society, 182
Malformation in the Uterus not Produced by Mental Influence of the Mother, ------------ 519
Medical Association, British, --- 642
Money Receipts, -----------719, 785
Medicine and Surgery, Photograph Review of, ------------ 716
Muscular Exercise, Influence of on the Urine, ------------- 712
Milk, Secretion of Restored by Electricity, ---------------- 718
Mouth, Deformity of the. Diffenbach's Operation, ---------- 692
Medical Journals Changes in, --- 182
Malt Extract. By Albert E. Ebert, ------------------- 755
Muscular Exercise, The Physiological Effect of, ------------ 763
Malarial Toxæmia, Acute, and the Pathology of Diseases of Malarial Origin, ----------- 766

Nebraska State Medical Society, 50
New York Academy of Medicine, 254
National Medical Journal, ----- 313
Nephrotomy, ------------------ 317
New Mercy Hospital, ---------- 312
Notes from the Wards of Mercy Hospital. By C. W. Earle, M.D., 277
Naval Hygiene. By Jos. Wilson, Surgeon, U. S. N., ---------- 584
Neuralgia of the Jawbones, ----- 633

"Os and Cervix Uteri." By Noble Holton, M.D., ------------- 1
Opium Eating, Excessive, ------ 60
Ovariotomy—Sequel of Dr. Russell's Case. By J. F. Kelsey, M.D., --------------------- 133
Obstetric Aphorisms. By J. G. Swayne, M.D., ------------ 178
Obstetrical and Gynæcological Section of the 48d Congress of the German Physicians, etc., -- 234
Oxygen Gas as a Remedy in Disease. By A. H. Smith, M.D., 290, 625
Old University of Bologna. By F. H. D., ------------------ 288
Our Home Physician. By Géo. M. Beard, A.M., M.D., ------- 388

PAGE.
Ovariotomy—Twice on one Patient, ---------------------- 461
Obituary—Jos. S. Hildreth, M.D., 526
Ossification of the whole Muscular System, -------------- 522
Ovariotomy, a Case of. By M. A. Chase, M.D., ------------- 534
Otology, Report on. By Saml. J. Jones, A.M., M.D., -------- 605
Ohio State Medical Society, ---- 710
Obstetrics. By W. H. Byford, M.D 709
Opium in Metro Peritonitis. By Theodore Griffin, M.D., ------ 744
Occlusion of the Vagina with Retention of the Catamenia, A Case of, ------------------- 764
Opomorphia, ----------------- 770

Plastic Operations. By David Prince, M.D., ------------- 16
Prescriptions, Repeating. A Remedy for. By T. Griffin, M.D., - 23
Permangnate of Potassia, -------. 32
Phosphorus and its Compounds, - 57
Pepsine, a New Preparation of. By Edward Long, L.A.H., ---- 176
Prof. Bigelow's Reply to Prof. Gunn, --------------------- 183
Phosphorus, an Antidote to, ---- 252
Pyemia, Treated with Sulphite of Soda. By O. W. Sadler, M.D., 198
Puerperal Fever in Cook County Hospital, ----------------- 218
Phthisis, Arsenic in, ---------- 250
Prurigo Treated by Ointment of Iodoform, ---------------- 317
Professional Quarrels ---------- 390
Physiology. By Wm. T. Lusk, M.D., --------------------- 446
Pancreatic Secretion, ---------- 459
Professional Changes, ---------- 524
Physiology of Man. By Austin Flint, Jr., M.D., ------------ 583
Physicians and Druggists in America, ------------------ 639
Placenta Previa and Double Births, -------------------- 639
Plaster-Paris, Use of in Fractures. By R. G. Bogue, M.D., ------ 617
Practice of Medicine. By T. H. Tanner, M.D., F.L.S., -------- 639
Puerperal Convulsions, Tedious Labor. By N. S. Davis, M.D., 679
Pepsin, The Use of, ----------- 764
Pulmonary Consumption, Treatment of, ------------------- 765

PAGE.
Pulmonary Fetor, 787
Prizes, Fisk Fund, 776

Quackery and the Press, 393

Reply to Prof. Bigelow. By Prof. Gunn, 98
Relapsing Fever in Edinburgh, 289
Relations of Physical Forces to Disease. By F. H. Blackman, 267
Remarkable Fall. By Henry Shimer, 289
Renal Diseases. By W. R. Basham, M.D., 450
Renal Disease, Diagnosis of, 456
Rectum, Physical Exploration of the. By Wm. Bodenhamer, A.M., M.D., 640
Report of Delegates to the Convention of College of Pharmacy, 758
Removal of Iron Stains from Fabrics, 776

Svapnia. Letter from John M. Bigelow, 30
Spinal Cord, Modifications which take place in. By A. Vulpian, 77
Stenocardia. Lectures of Prof. Oppolzer, 171
Spring and Summer Medical Instruction, 180
Sensibility of the Nervous Centres, Errors in Relation to. By Brown-Sequard, 139
Scarlet Fever, Clinical Lectures on. By Sir Wm. Jenner, M.D., 221
Shock, 461
Saccharine Diabetes, Bromide of Potassium in, 461
Societies, Proceedings of, 451
Strabismus. By H. W. Boyd, M.D. 465
Scapula, Excision of a, 522
Shaw's Med. and Surg. Case Book, 584
Surgery, Report on. By Moses Gunn, M.D., 544
Sick, the, 588
Stetho-Sphygmograph, the, 649
Supra-Uterine Pressure, 631
Strychnine as an Antidote for Chloral-Poisoning, 645
Sulpho-Carbolates are not powerful direct Antiseptics, 635
Salivary Calculus, Removal of. By Chas. M. Clark, M.D., 622
Supra-Renal Capsules, 700

Tetanus, Following Compound Fracture of the Leg, Recovery, 193

PAGE.
Tabes Mesenterica, With Cases. By F. K. Bailey, M.D., 274
Therapeutics, Modern. By Geo. H. Napheys, A.M., M.D., 310
Typhoid Fever. By A. Given, M.D. 321
Toland Medical College. Letter from Faculty of, 366
Tonsils, Enlarged, Removed without Cutting, 394
Tobacco, Smoking, Effects of on Children, 365
Tetanus Produced by the Administration of Quinia, hypodermically, 460
Traumatic, Irideremia and Aphakia. By C. Hixson, M.D., 564

University of Michigan. Letter from Faculty of, 368
Uterus, Inverted, New Operation 516
Uterus, Inverted, treated Successfully by Elastic Pressure. By W. H. Byford, A.M., M.D., 481
Urine, A Guide to the Examination of. By J. W. Legg, M.D., 640
Uterus, Catarrh of the, 638
Uterus, Cancer of, 696
Uric Acid in the Blood, To Detect 743
Uterine Contraction, Force of, 760

Vegetable Parasites, Report on. By N. S. Davis, M.D., 70
Vital Statistics, 520
Vaccinal Syphilis. By W. B. Davis, M.D., 557
Veratrum Viride in Pneumonia, 637
Vesicant, A New, 650
Vaginal Ovariotomy, 632
Variola. By E. A. Carpenter, M.D. 674
Vaccination for Whooping-cough, with Clinical Cases. By Z. C. McElroy, M.D., 734

White Blood Corpuscles, Relation of to Suppuration Inflammation. By A. Henocque, 201
West, Dr. Charles, Something in Regard to, 212
Woman's Hospital Medical College of Chicago, 585
Wabash Valley Æsculapian Soc'y 567
Wisconsin State Medical Society, 573
Woman's Hospital Medical College, A lecture Introductory to the Course of Instruction in the. By W. H. Byford, A.M., M.D. 721

